# Assessing the calibration in toxicological in vitro models with conformal prediction

**DOI:** 10.1186/s13321-021-00511-5

**Published:** 2021-04-29

**Authors:** Andrea Morger, Fredrik Svensson, Staffan Arvidsson McShane, Niharika Gauraha, Ulf Norinder, Ola Spjuth, Andrea Volkamer

**Affiliations:** 1 In Silico Toxicology and Structural Bioinformatics, Institute of Physiology, Charité Universitätsmedizin, Berlin, Germany; 2 Alzheimer’s Research UK UCL Drug Discovery Institute, London, WC1E 6BT UK; 3 Department of Pharmaceutical Biosciences and Science for Life Laboratory, Uppsala University, 751 24 Uppsala, Sweden; 4 Division of Computational Science and Technology, KTH, 100 44 Stockholm, Sweden; 5 Dept. Computer and Systems Sciences, Stockholm University, Box 7003, 164 07 Kista, Sweden; 6 MTM Research Centre, School of Science and Technology, Örebro University, 70 182 Örebro, Sweden

**Keywords:** Toxicity prediction, Conformal prediction, Data drifts, Applicability domain, Calibration plots, Tox21 datasets

## Abstract

Machine learning methods are widely used in drug discovery and toxicity prediction. While showing overall good performance in cross-validation studies, their predictive power (often) drops in cases where the query samples have drifted from the training data’s descriptor space. Thus, the assumption for applying machine learning algorithms, that training and test data stem from the same distribution, might not always be fulfilled. In this work, conformal prediction is used to assess the calibration of the models. Deviations from the expected error may indicate that training and test data originate from different distributions. Exemplified on the Tox21 datasets, composed of chronologically released Tox21Train, Tox21Test and Tox21Score subsets, we observed that while internally valid models could be trained using cross-validation on Tox21Train, predictions on the external Tox21Score data resulted in higher error rates than expected. To improve the prediction on the external sets, a strategy exchanging the calibration set with more recent data, such as Tox21Test, has successfully been introduced. We conclude that conformal prediction can be used to diagnose data drifts and other issues related to model calibration. The proposed improvement strategy—exchanging the calibration data only—is convenient as it does not require retraining of the underlying model.

**Supplementary Information:**

The online version contains supplementary material available at 10.1186/s13321-021-00511-5.

## Introduction

Machine learning (ML) methods are ubiquitous in drug discovery and toxicity prediction [1, 2]. In silico toxicity prediction is typically used to guide toxicity testing in early phases of drug design [3]. With more high-quality standardised data available, the (potential) impact of ML methods in regulatory toxicology is growing [4]. The collection of available toxicity data is increasing, thanks in part to high-throughput screening programs such as ToxCast [5] and Tox21 [6, 7], but also with public-private partnerships such as the eTOX and eTRANSAFE projects, which focus on the sharing of (confidential) toxicity data and ML models across companies [8, 9]. In any case, no matter which underlying data and ML method is used, it is essential to know or assess if the ML model can be reliably used to make predictions on a new dataset.

Hence, validation of ML models is crucial to assess their predictivity. Several groups investigated random vs. rational selection of optimal test/training sets, e.g. using cluster- or activity-based splits, with the goal of better reflecting the true predictive power of established models [10–14]. Martin et al. [11] showed that rational selection of training and test sets—compared to random splits—generated better statistical results on the (internal) test sets. However, the performance of both types of regression models on the—artificially created—external evaluation set was comparable.

Thus, further metrics to define the applicability domain (AD), the domain in which an ML classifier can reliably be applied [15–21], are needed. Besides traditional metrics accounting for chemical space coverage, Sheridan [20] discussed uncertainty prediction regression models, fitted with the activity prediction errors as labels and diverse AD metrics as descriptors (e.g. accounting for variation among RF tree predictions, predicted activity ranges with different confidence, or similarity to nearest neighbours). Since in classification models, the response/activity is a categorical value, only the chemical space remains to define the AD. Mathea et al. [15] categorised the available methods into novelty and confidence estimation techniques. The former consider the fit into the underlying chemical descriptor space as a whole, whereas the latter focus on the reliability of predictions, i.e. data points may be well embedded in the descriptor space but abnormal regarding their class label.

A popular method for confidence estimation is conformal prediction (CP), which has in recent years been widely applied in the drug discovery and toxicity prediction context [15, 22]. In CP, ML models are trained, and with the help of an additional calibration set (inductive conformal prediction [23]), the predictions are calibrated, i.e. ranked based on previously seen observations, resulting in so-called conformal p-values or simply p-values (not to be confused with statistical p-values from hypothesis testing). The design of the CP statistical framework guarantees that the error rate of the predictions will not exceed a user-specified significance level. The control of this significance level makes CP advantageous compared to traditional confidence estimation methods, such as distance from the decision boundary, or ensemble models [15].

ML algorithms rely on the assumption that the probability distribution of the training data and test data are *I*.*I*.*D.* (independent and identically distributed). For conformal prediction, a slightly weaker assumption in the form of exchangeability is assumed for producing well-calibrated models [24]. This assumption is nevertheless not always fulfilled, especially when training and test data come from different sources. For example, data drifts were observed between training and test data of the USPS (handwritten digits) and the Statlog Satellite (satellite image) datasets [25]. Similar observations were made in the toxicity prediction context when applying androgen receptor agonism CP models trained on publicly available data to an industrial dataset [26]. Some efforts to look at data exchangeability include studies using martingales to uncover exchangeability issues in an online setting [25].

In this work, we explored how the above described concepts of conformal prediction can be used to assess the quality of the model calibration when trained and applied on various toxicological in vitro datasets or subsets. For this purpose, the freely available Tox21 datasets [27], initially prepared for a data challenge to encourage model building and benchmarking toxicity prediction, were used. We show that conformal prediction allows us to identify data drifts between the Tox21 datasets, and we also propose strategies to mitigate this.

## Data and methods

In this section, first the used Tox21 datasets are introduced. Second, the general conformal prediction framework along with aspects such as aggregation, evaluation and strategies to improve the calibration are described. Finally, the set-up and the individual computational experiments of this work are explained, including a reference to code and data availability.

### Data collection, preprocessing and encoding

#### Tox21 datasets

The investigations in this work were performed on the freely available Tox21 datasets [27]. They consist of approximately 10,000 chemicals, which were tested on up to 12 endpoints of the nuclear receptor (NR) and stress response (SR) pathways. As the dataset was released in a challenge setting, the three subsets were chronologically published to the Tox21 Data Challenge participants: Tox21Train for training the models, Tox21Test as an intermediate set for the leaderboard to check the performance (and for participants to improve their models), and Tox21Score as the final dataset to determine the best performing models. The respective datasets were downloaded from the US National Center for Advancing Translational Sciences [28] on January 29th, 2019. Each compound was provided in sdf-format together with a binary value (0/non-toxic, or 1/toxic) for each of the 12 endpoints (*X* if no assay outcome was available for the compound). Note that throughout this manuscript, the Tox21 datasets are, consistently, referred to as Tox21Train, Tox21Test and Tox21Score, this should not be confused with additional training and test set splits necessary for the ML/CP model set-ups.

#### Data preprocessing

The datasets were standardised as described in Morger et al. [26]. Briefly, the IMI eTox standardiser tool was applied to discard non-organic compounds, to exert certain structure standardisation rules, to neutralise, and to remove salts [29]. Before and after applying the standardisation protocol, compounds with duplicate InChIs (IUPAC International Chemical Identifiers [30]) but disagreeing labels were discarded. Furthermore, remaining mixtures and fragments with less than four heavy atoms were removed. The numbers of data points available per dataset and endpoint after standardisation are presented in Table 1. The corresponding numbers before standardisation can be found in the Additional file 1: Table S1.

**Table 1:** Number of compounds (separated as actives and inactives) available per Tox21 dataset and endpoint after standardisation. The full names for the endpoints are adopted from Huang et al. [31]

Endpoint	Tox21Train		Tox21Test		Tox21Score	
	Actives	Inactives	Actives	Inactives	Actives	Inactives
Aryl hydrocarbon receptor (NR_AhR)	933	6687	29	236	71	506
Androgen receptor, full length (NR _AR)	373	8370	3	282	11	549
Androgen receptor, ligand binding domain (NR_AR_LBD)	295	7742	4	242	8	543
Aromatase (NR_Aromatase)	338	6362	18	192	36	466
Estrogen receptor, full length (NR_ER)	901	6290	27	231	49	441
Estrogen receptor, ligand binding domain (NR_ER_LBD)	419	7763	10	270	20	548
Peroxisome proliferator-activated receptor gamma (NR_PPAR)	204	7414	15	245	31	543
Nuclear factor (erythroid-derived 2)-like 2/antioxidant responsive element (SR_ARE)	1032	5653	47	181	89	433
ATAD5 (SR_ATAD5)	322	8179	25	240	36	554
Heat shock factor response element (SR_HSE)	386	7233	10	250	19	558
Mitochondrial membrane potential (SR_MMP)	1094	5719	38	195	56	457
p53 (SR_p53)	515	7542	28	234	40	543

#### Compound encoding

Converting molecules into numerical data was performed using the signature molecular descriptor [32, 33], using the program CPSign [34] version 0.7.14. The signature descriptor has been used extensively in previous QSAR studies [35–37]. In brief, the signature molecular descriptor enumerates all fragments of a molecule using a specified number of atomic bonds, often referred to as height, here using height 1 to 3 (e.g., height 1 creates fragments containing a center atom and all its one-bond connected atoms). This descriptor is often extremely sparse as there is a large number of fragments in a dataset and each molecule contains only a small set of these fragments. Herein, the count of each fragment was used; it is also possible to use a bit-type vector, where 0/1 indicates whether the fragment is present or not. The composition of the training set and hence the number of descriptors is different per endpoint. On average 36,721 (± 2363 std) fragments were defined per endpoint in the Tox21Train set, whereas the signatures for Tox21Test and Tox21Score are based on the fragments in Tox21Train.

### Modelling

#### Conformal prediction

**Fig. 1:**
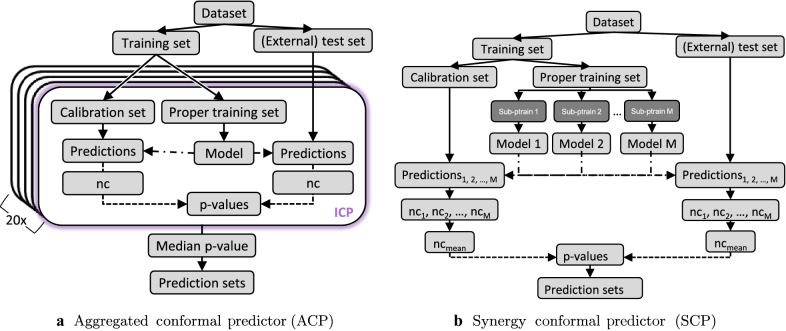
Inductive conformal predictor (ICP) and the aggregated conformal prediction methods used in this study. **a** Aggregated conformal prediction (ACP) and ICP (box with purple edge): The dataset is split into a training set and a test set. The training set is further split into a proper training set to train the model and a calibration set. The predictions made for the test set compounds are used to calculate nonconformity scores (nc) and compared to nonconformity scores in the calibration set to calculate p-values and generate prediction sets. In ACP, multiple models are trained and calibrated with randomly selected proper training and calibration sets, and p-values from these are averaged. **b** Synergy conformal prediction (SCP): In order to ensure a uniform distribution of p-values, SCP averages the nonconformity scores instead. Multiple models are trained on (subsets of) the proper training set and with each model predictions are made for the test set and for a fixed calibration set

Conformal prediction (CP) is a statistical framework, which provides means for confidence estimation [15, 38]. The baseline conformal predictor is the computationally efficient inductive conformal predictor (ICP) [23] (indicated in purple in Fig. 1a). An ICP operates on the output from an underlying model. To allow calibration of the outputs, the training set is divided into a proper training set and a calibration set. An underlying model, most often a machine learning model, is fitted on the proper training set, predictions are made for both the test and the calibration set compounds, and transformed into so-called nonconformity scores. In a binary Mondrian setting [38, 39], for each test compound two p-values are calculated, one per class, by comparing the outcome of each instance with the outcomes of the corresponding calibration set compounds. Given the two p-values and a predefined significance level $\epsilon = 1 - \text {confidence level}$, a prediction set is calculated. The prediction set contains all class labels, for which the p-value is larger than the significance level. For more information on conformal prediction, see Alvarsson et al. [40] and Norinder et al. [41].

#### Aggregated conformal prediction methods

To reduce the variance in efficiency of ICPs, multiple conformal predictors can typically be aggregated [42, 43] (see Fig. 1). In the commonly used aggregated conformal prediction (ACP) [43] aggregation method, the training set is randomly split n times into a proper training set and a calibration set, with which n ICPs are trained and calibrated (Fig. 1a). The p-values resulting from the different ICPs are then averaged. While the consolidation of multiple models stabilises the predictions, a uniform distribution of the p-values is not necessarily observed after their averaging [42].

The influence of ACPs on the calibration can be analysed by additionally incorporating the recently developed synergy conformal prediction (SCP) method (Fig. 1b) [44]. In the SCP, one fixed calibration set is randomly selected, and the proper training set is split into n subsets to train multiple sub-models. Note that the analysis of other options to build an SCP, e.g. training several models using different ML algorithms on the same (sub)set, is out of scope for this work. The predictions made with every sub-model are aggregated before calculating the p-values and prediction sets. A fixed calibration set reduces the number of available training compounds, but the needlessness of averaging p-values ensures a uniform distribution of the latter and hence leads to theoretically valid models [44].

#### Model evaluation

CP models are typically evaluated by their validity and efficiency [15]. Validity, for a given significance level, is defined as the ratio of prediction sets that contain the true label. The efficiency of a model is a way to measure the information content of the model, and we herein use the most widely used efficiency metric: ratio of single label sets at a given significance level. In binary CP, the possible prediction sets are {$\emptyset$}, {0}, {1} and {0,1}, where only the {0} and {1} (i.e. single label sets) are informative, and ‘empty’ and ‘both’ sets are uninformative in a sense. Thus, the fraction of single label sets should be maximised for best efficiency.

### Model calibration

When evaluating the predictive performance on a test set, deviations from the underlying assumption that all data come from the same distribution will lead to predictions that are invalid and hence the results might be misleading. In this work, we use calibration plots to identify deviations from acceptable levels of calibration, and also discuss potential mitigation strategies.

#### Assessing model calibration

**Fig. 2:**
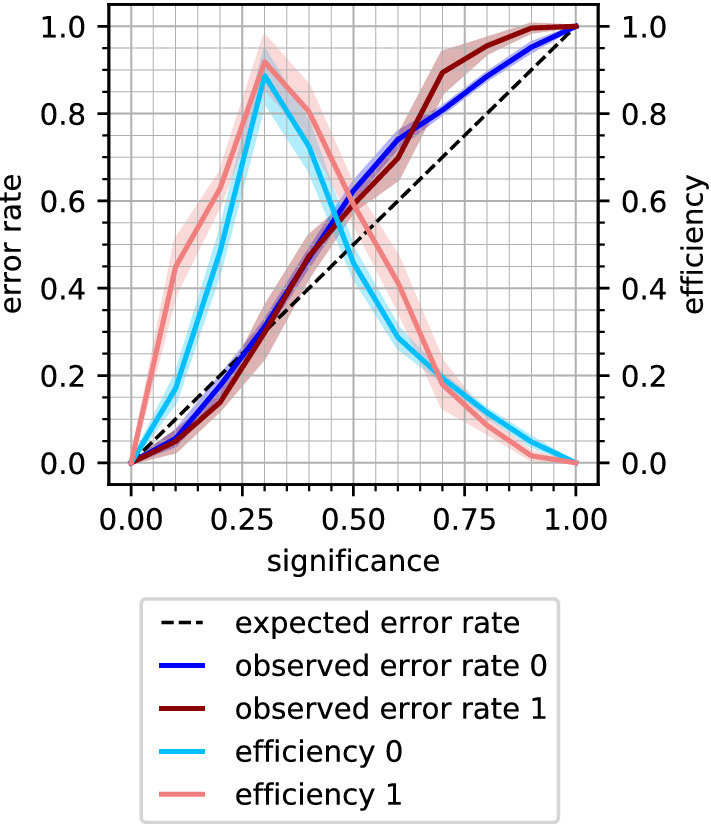
Calibration and efficiency plot. The dark lines show the mean error rate for the active (dark red) and inactive (dark blue) compounds. For a well-calibrated model, the error rate ideally follows the dashed diagonal line. The light coloured lines illustrate the mean efficiencies expressed as ratio of single label sets for the active (light red) and inactive (light blue) compounds. The shaded areas indicate the respective standard deviations within the fivefold CV. Class 0: inactive compounds, class 1: active compounds

In a conformal prediction setting, the observed error rate of predictions is theoretically proven to not be larger than the specified significance level. In return, any deviations between these values may indicate data drifts (or other causes for the deviations, such as a too small test set). The level of calibration can be visualised in a so-called calibration plot, where the observed error rate (y-axis) is plotted versus the significance level (desired error rate, x-axis). For valid (well-calibrated) models the values should lie on the diagonal line. Deviations from this behaviour signals deviations from perfect calibration. We also include efficiency in the plot, calculated as the fraction of single-class predictions. These plots, from hereon called calibration and efficiency plots (CEPs), were used in this work to assess the model calibration and efficiency (see Fig. 2). As a measure of the level of calibration, we use the root-mean-square deviation (RMSD) between the specified significance and the observed error rate.

#### Model update strategies

**Fig. 3:**
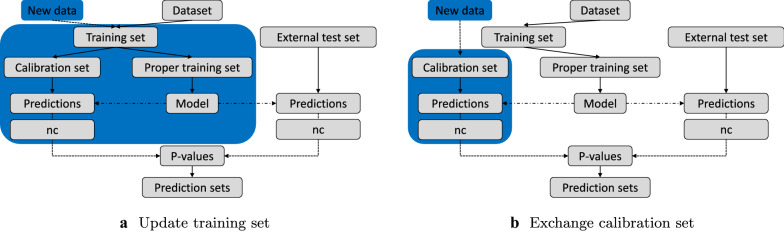
Model update strategies analysed to improve calibration. **a** Update training set: The whole training set is updated with new data. This involves retraining a new model. **b** Exchange calibration set: Only the calibration set is updated with new data. Models can hereby be re-calibrated without training a new model

In a setting where a model has been trained but new data on the same or a similar endpoint is made available, it is interesting to consider how the new data should be utilised in order to improve primarily the level of calibration but also the efficiency. We investigated two update strategies, see Fig. 3. The first strategy included updating the whole training set with new data followed by subsequent retraining of the model (see Fig. 3a). In the second strategy, the proper training set was kept and only the calibration set was exchanged with more recent data (see Fig. 3b).

### Study design

**Fig. 4:**
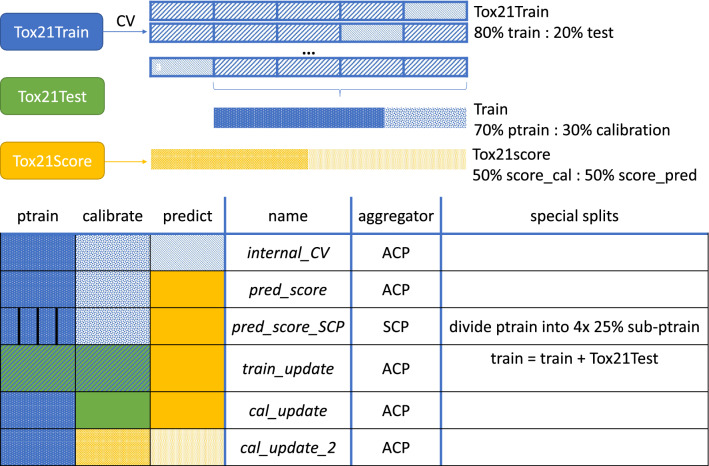
Overview of the experiments discussed in this work. Top: Splitting of Tox21 data into (proper) training, calibration and test set. Bottom: Data for training, calibration, and prediction as well as aggregator used in the specific experiments.

In this work, six different CP experiments were explored as illustrated in Fig. 4 and Table 2. The first experiment consisted of a cross-validation (CV) using ACP on the Tox21Train dataset (*1-internal_CV*), the second comprised predictions with the CV-models from experiment 1 on the Tox21Score dataset (*2-pred_score*). In the third experiment, the influence of ACP on the calibration was assessed by training an SCP model on Tox21Train and predicting Tox21Score (*3-pred_score_SCP*). Finally, in the last three experiments, the model update strategies to improve the calibration were evaluated (see Fig. 3). Thus, in experiment 4 the training set was updated (*4-train_update*) and the model retrained, while in experiment 5 and 6 only the calibration set was updated (*5-cal_update* and *6-cal_update_2*).

**Table 2:** Overview of the experiments discussed in this work. Note that all splits were performed randomly stratified

Nr.	Name	Explanation
1	*internal_CV*	A fivefold CV, training one ACP per fold, is performed on the Tox21Train dataset and internally evaluated on the respective hold out data.
2	*pred_score*	Using the CV-models trained within the above described CV, the Tox21Score data are predicted.
3	*pred_score_SCP*	The same CV splits are applied as described above. The training set is then split into a fixed calibration set and four proportionate sub-proper training sets. For each of the four corresponding sub-proper training sets, an ML model is trained. Predictions are made for Tox21Score (and the calibration set compounds) with every model; the four nonconformity scores (ncs) are averaged before calculating the p-values.
4	*train_update*	The training set from the CV is combined with the Tox21Test set. This updated training set is then split into proper training and calibration set to train new ACP models for the CV set-up. Tox21Score data are predicted with the new models.
5	*cal_update*	The CV-models from experiment 1 are used, but the calibration is updated with the Tox21Test data to predict Tox21Score.
6	*cal_update_2*	The CV-models from experiment 1 are used, but the calibration is updated with 50% of Tox21Score data. The other 50% of Tox21Score are predicted. In every fold of the CV, Tox21Score is split in two equal subsets.

The individual experiments were conceptualised in a way that the proper training sets were consistent across all experiments (where applicable). A fivefold CV was implemented, not only for internal validation (*1-internal_CV*), but conserved for all experiments. Hence, the selected data per CV loop of a fivefold CV were retained for all trained models (i.e. in the *1-internal_CV*, *4-train_update* and *3-pred_score_SCP* experiments). Specifically, the indices of the training compounds were saved, so that the same training sets could be used for the subsequent experiments. This ensures that the results from the different experiments can be directly compared. For the ACP model, 20 aggregated ICPs were used with 30% (of the training set) set aside as a calibration set and 70% as a proper training set. For the *3-pred_score_SCP* experiment (using SCP, see Fig. 1a), the training set was split into a fixed 30% calibration set and the proper training set divided into four equally sized partitions. For the *4-train_update* experiment, the training set was first updated with the Tox21Test dataset and then split into calibration and proper training set using the above described ratios. For the two experiments updating the calibration set, the same trained CV-model from *1-internal_CV* was calibrated with only the Tox21Test dataset (*5-cal_update*) and in the last experiment (*6-cal_update_2)* replacing the calibration examples with 50% randomly stratified split Tox21Score data.

SVM models were trained using the Scikit-learn Python library [45] version 0.23.2 with an RFB kernel, C = 50, $\gamma$ = 0.002) [37]. For conformal prediction, the nonconformist Python library [46] was used with margin error function, Mondrian condition [38, 39] version 2.1.0. For ACP, p-values were aggregated by median (see [42]), for SCP the nonconformity scores were averaged before calculating p-values.

### Code and data availability

A GitHub repository associated with this work is available at https://github.com/volkamerlab/cptox21_manuscript_SI. It contains the signature fingerprints for all pre-processed datasets as well as example code to demonstrate how the different ACP experiments were performed. The repository also provides the result files containing the respective measures for all experiments, from which the CEPs and boxplots can be generated. The SCP code is available from the original SCP repository by Gauraha et al. [44, 47].

## Results and discussion

The aim of this study was to assess the level of calibration between the initial release of the Tox21Train data and the subsequently released Tox21Score data using conformal prediction (experiments 1–3). In follow-up experiments, we also investigated two model update strategies for incorporating the Tox21Test data (experiments 4–6). An overview of the error rates and efficiencies at significance level 0.2 for all experiments is provided in the Additional file 1: Table S2.

### Experiment 1: Cross-validation on the Tox21Train datasets

Before applying a model to external data, it needs to be validated by ensuring that the model is internally well calibrated. Hence, in a first experiment (*1-internal_CV*), models were built in a fivefold CV scenario on the Tox21Train datasets. The models for the 12 Tox21 endpoints were internally valid with a mean error rate of 0.17 (± 0.01 std) at significance level 0.2, as well as a high mean efficiency of 0.77 (± 0.13 std).

**Fig. 5:**
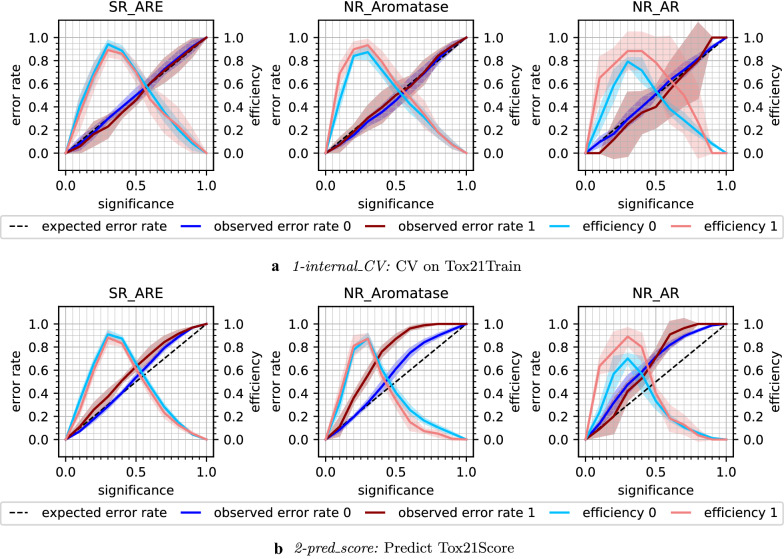
CEPs for models trained on Tox21Train and subsequent internal cross-validation (**a**) and predictions on Tox21Score (**b**). CEPs for a selection of three example endpoints (SR_ARE, NR_Aromatase, NR_AR). Class 0: inactive compounds, class 1: active compounds. For a detailed explanation of all the components in the CEP, see Fig. 2

The error rates and efficiencies over all significance levels (mean and std of the five CV folds per model) are illustrated in CEPs (Fig. 5a) for three example endpoints (namely SR ARE, NR_Aromatase and NR_AR; the remaining CEPs are shown in the Additional file 1: Figure S1). While the models are overall well calibrated, i.e. the observed error rates follow the diagonal line in the CEPs, and the standard deviations between the individual runs are low, there are a few outliers. The high variance (see shaded areas in the CEPs) for the active compounds and the low efficiency for NR_AR reflect the observations in the Tox21 data challenge that NR_AR was one of the most difficult targets to model and has, with 387 active and 9201 inactive compounds, the lowest active compound rate after NR_PPAR$\gamma$ and NR_AR_LBD [31]. The well-calibrated models were ready to be applied to external data which stem from the same distribution as the training data.

### Experiment 2: Model performance on the Tox21Score datasets

To investigate how well the CP models from the cross-validation perform on an external dataset, predictions were made for the Tox21Score data (*2-pred_score*). A mean error rate at significance level 0.2 of 0.31 (± 0.12 std) was achieved. The efficiency dropped only slightly to 0.72 (± 0.14 std). The deviations from the diagonal line in the CEPs (Fig. 5b, Additional file 1: Figure S2) for most of the endpoints indicate that the calibration of the models was poor when predicting Tox21Score.

Note that predictions were also made for the Tox21Test compounds (shown in the Additional file 1: Figure S3 only, referred to as *pred_test*). This set-up was similar to the intermediate setting in the Tox21 challenge, where predictions on Tox21Test were decisive for the leaderboard. The mean error rate at significance level 0.2 over all endpoints was higher than expected (0.26 ± 0.11 std). So, the models were not well-calibrated for predictions on Tox21Test. The mean efficiency was 0.70 (± 0.15 std), i.e. similar to 2-*pred_score results*. The poor calibration for the predictions on both (external) datasets is an indication that the Tox21Score and the Tox21Test data might come from a different distribution than the Tox21Train data.

### Experiment 3: Influence of aggregation method on the calibration

Reasons for poor calibration can be the difference between the distribution of two datasets, but also the data set size (discussed later) or the aggregation strategy for the conformal predictor (here ACP). From a theoretical perspective, the use of ACP can affect the calibration, as ACPs have not been proven to be always valid [42]. In ACP, the p-values from all ICPs are aggregated, which in theory could result in a non-uniform distribution. To rule out that the use of ACP is the (main) reason for the poor calibration, the recently developed SCP aggregation method was applied. In the SCP framework (see Fig. 1b), nonconformity scores are averaged before calculating the p-values, which are the basis for the calibration. This aggregation method has been shown to be theoretically valid [44].

**Fig. 6:**
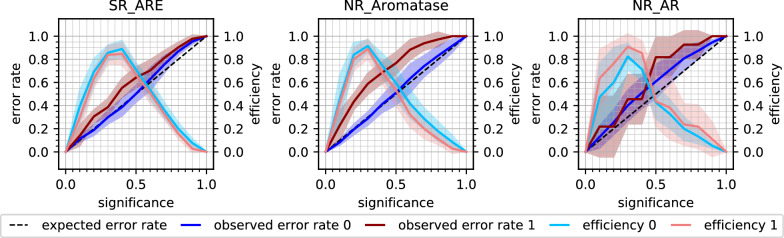
Results from experiment *3-pred_score_scp:* SCP models were trained on Tox21Train and predictions made for Tox21Score. CEPs are shown for a selection of three example endpoints (SR_ARE, NR_Aromatase, NR_AR). Class 0: inactive compounds, class 1: active compounds. For a detailed explanation of all the components in the CEP, see Fig. 2

Applying SCP improved the calibration on the Tox21Score dataset (*3-pred_score_SCP*), the mean error rate decreased to 0.27 (± 0.12 std) and the mean efficiency at significance level 0.2 was 0.73 (± 0.13 std). The error rates and efficiencies over all significance levels are shown in the CEPs in Fig. 6 for the SR_ARE, NR_Aromatase, and NR_AR endpoints and in the Additional file 1: Figure S4 for all 12 endpoints. It is especially noticeable that the calibration curves in the CEPs became less sigmoidal for many endpoints—such sigmoidal curves have typically been observed for ACPs [42, 44]. The sigmoidal shape is unfavourable from a theoretical perspective as it means that the model is poorly calibrated at low and high significance levels, but may be less problematic in an application context since the error rate is typically over-conservative at lower (i.e. relevant) significance levels. One drawback of SCP is the fixed calibration set, which means that part of the training set information is never used for training. Together with the smaller proper training set partitions, this can lead to less efficient predictions. This can be seen in the relatively large standard deviations of the error and efficiency rates in the CEPs (Fig. 6 and Additional file 1: Figure S4). For this reason, and since ACP is commonly used in literature, which makes the outcomes more comparable with work by other scientists, ACP was used for the subsequent experiments.

Summarising the results from experiment 1–3, it was concluded that the Tox21Test and Tox21Score data may originate from slightly different distributions than the Tox21Train data. This could be explained by knowing that the three Tox21 datasets were created (screening of compounds) at different stages. For the Tox21Train set, the actual “Tox21 10K dataset” [31] was used, for which data had been available at the start of the challenge. The Tox21Test dataset is part of the LOPAC$^{1280}$ (Library of Pharmacologically Active Compounds) dataset, which was used to validate the Tox21 assays [31, 48]. The Tox21Score data were separately provided by the EPA and only screened during the challenge [31]. So-called data or assay drifts typically occur over time or when moving towards a different chemical space [49].

### Experiment 4: Effects on calibration by updating the training set

When the model is not well calibrated for the predictive task and newer data are available, one would intuitively combine these additional data (i.e. Tox21Test) with the previous training data (i.e. from *1-internal_CV*), train a new model, and use it to predict Tox21Score (*4-train_update*). Following this strategy, the mean error rate over the 12 endpoints dropped to 0.23 (± 0.06 std) compared to the predictions with the model built on the Tox21Train data (*2-pred_score*, 0.31 ± 0.12 std). The mean efficiency at significance level 0.2 (0.71 ± 0.15 std) was in a similar range as with the original training set (0.72 ± 0.14 std). Thus, the updating of the training set and retraining the model led to a small improvement in calibration (see CEPs in Additional file 1: Figure S5). One reason why we observed only a minor improvement of the calibration could be the sizes of the two datasets. The update set (254 ± 22 compounds) is small compared to the original training set (7647 ± 692 compounds) and has thus a lower influence on the new model. Furthermore, this strategy involves additional computational resources and the data of the previous model needs to be available for retraining.

### Effects on calibration by updating the calibration set

#### Experiment 5: Replace the calibration set with observations from Tox21Test

An alternative to updating the whole training set is to replace only the calibration set with the more recent data. This comes with the additional advantage that the calibration set can be renewed even if the training data are unavailable.

**Fig. 7:**
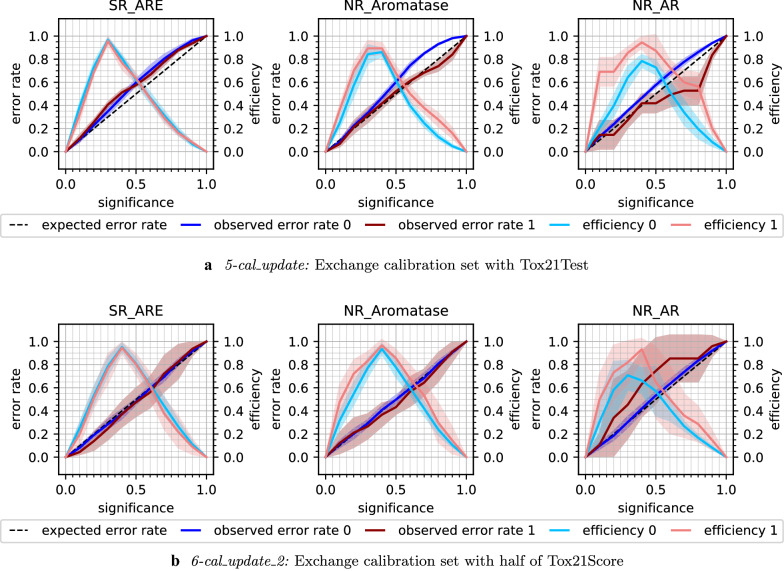
Updating the calibration set with more recent data from Tox21Test (**a**) or Tox21Score (**b**). CEPs for a selection of three example endpoints (SR_ARE, NR_Aromatase, NR_AR). Class 0: inactive compounds, class 1: active compounds. For a detailed explanation of all the components in the CEP, see Fig. 2

Updating the calibration set did result in a lower mean error rate of 0.21 (± 0.05 std) for the predictions on Tox21Score (*5-cal_update*). The mean efficiency at significance level 0.2 dropped to 0.51 (± 0.18 std). The loss in efficiency at low significance levels can be observed in the CEPs (Fig. 7a and Additional file 1: Figure S6), where the peak in efficiency is shifted towards higher significance levels. In the same CEPs, the improved calibration can be seen in the lower error rates. For six endpoints, when considering inactive compounds, or 11 endpoints, with regard to active compounds, even overconservative validity, i.e. a lower than expected error rate was achieved.

#### Experiment 6: Exchange the calibration set with half of Tox21Score

The chronological order of how the experimental data were produced is given by the Tox21 challenge organisers [31]. However, it is not clear if the compounds contained in Tox21Score (and Tox21Test) were really developed later than those in Tox21Train. For a ‘perfect’ calibration, it is required that the calibration and the test set stem from the same distribution. To simulate this, a second updating experiment, i.e. *6-cal_update_2*, was implemented. While still using the same proper training set as for the former experiments, the updated calibration set was created from Tox21Score. In every of the five (original) CV folds, 50% of Tox21Score was (randomly stratified) selected to constitute the calibration set while the other 50% of Tox21Score was used as test set. With this set-up, calibration and test set originate from the same distribution. This was also reflected in the mean error rate of 0.18 (± 0.01 std) at significance level 0.2, which was in a similar range as for the *1-internal_CV* with the original calibration set (0.17 ± 0.01 std). Similar to the previous updating experiment *5-cal_update*, the efficiency decreased to 0.50 (± 0.17 std) at significance level 0.2. Note that also the size of the calibration set was similar to the former *5-cal_update* experiment, as the Tox21Score set contains roughly twice as many compounds (551 ± 35) as Tox21Test (254 ± 22). On the other hand, by using half of Tox21Score for calibration, only the other half of the compounds was available for use as test set. This could lead to higher variations, e.g. in the error rate, especially for datasets with few test compounds. Such an example is shown for the NR_AR endpoint, for which Tox21Score only contains 11 actives. The standard deviation (shaded area) for the error rate and efficiency of the active compounds (red) increased compared to the *5-cal_update* experiment (Fig. 7). For the other two example endpoints in Fig. 7b (SR_ARE and NR_Aromatase), the calibration improved considerably. Summarising, the CEPs in the Additional file 1: Figure S7 illustrate how the calibration improved after exchanging the calibration set with data from the same distribution as the test set, but also how the efficiency dropped compared to the *4-train_update* strategy (Additional file 1: Figure S5). The decrease in efficiency in the ‘cal_update’ experiments is undesired but can be an acceptable trade-off in cases where validity could be restored. However, it has to be noted that the *6-cal_update_2* scenario is not often practically applicable as the updated calibration data needs to be available before making predictions.

Ultimately, updating the calibration set has no impact on the applicability domain of the underlying model. Improved calibration level and lower efficiency rather indicate that more compounds outside the applicability domain might be detected and classified as ‘both’ prediction sets. Thus, applying the *5-cal_update* over the *4-train_update* strategy is mainly promising in a situation as described in this work where the number of available new compounds is limited.

### Quantification of the calibration for all experiments

The error rates (discussed above) depend on the desired significance level. In the calibration plot, the error rates are plotted over a range of significance levels. However, if the model will only be applied at a certain significance level, obtaining a good level of calibration at that significance level might be enough. But, if the calibration of the model is assessed from a theoretical perspective, all significance levels must be considered. This was illustrated for the individual experiments with the help of CEPs as discussed above. To have a comparable metric, the root-mean-square deviation (RMSD) over all significance levels (step-width 0.1) was calculated.

**Fig. 8:**
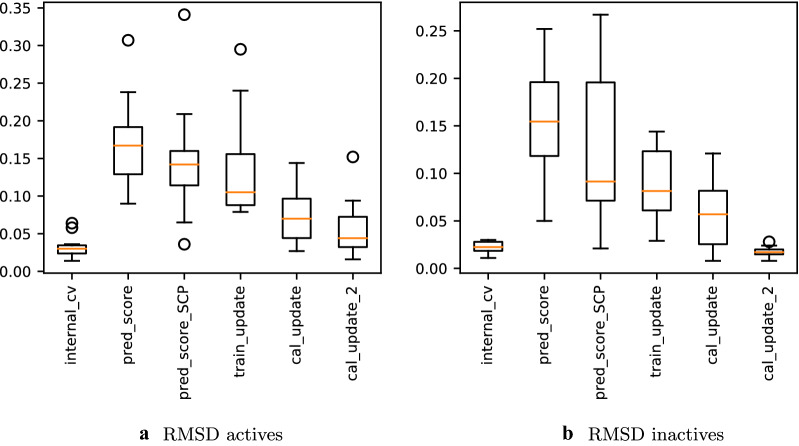
Box plots for the root-mean-square deviation (RMSD) between the expected and observed error rates for all 12 Tox21 endpoints compared amongst the different experiments are shown. On the left results for the active compounds (**a**), on the right for the inactive compounds (**b**) are plotted. Note that the y-axis ranges differ

Boxplots illustrating the RMSDs between observed and expected error rates over all endpoints are available in Fig. 8a for the active compounds and Fig. 8b for the inactive compounds, and show how the error rate deviations behave between the individual experiments. The mean RMSD values (overall, actives and inactives) for all experiments are provided in the Additional file 1: Table S3.

Clearly, the RMSD for the actives and inactives is low in the internal CV with Tox21Train (*1-internal_CV*) for most of the endpoints (mean overall RMSD: 0.022), while the deviations increased for the predictions on Tox21Score (*2-pred_score*, mean overall RMSD: 0.150). When using the SCP aggregation method (*3-pred_score_SCP*), the RMSD decreased for eight endpoints, albeit, only by a small amount (Fig. 8, mean overall RMSD: 0.121). Updating the training set (*4-train_update*, using ACP) led only to a small improvement of the mean RMSD of the active compounds (mean RMSD, actives: 0.135, Fig. 8a), while the improvement was more distinct for the inactive compounds (mean RMSD, inactives: 0.089, see Fig. 8b). When exchanging the calibration set with Tox21Test (*5-cal_update*), the RMSD decreased for 11 endpoints (except for SR_ARE, for which the calibration was already very good (overall RMSD SR_ARE, *1-pred_score*: 0.055) with the original calibration set). The mean overall RMSD (0.054) was, however, still not at the same level as for *1-internal_CV*. This can be attributed to overconservative validity, especially for the active compounds (see Additional file 1: Figure S6) which led to an increased RMSD for several endpoints. The overconservative validity almost disappeared when the calibration set was exchanged with data which are inherently exchangeable with the test set (*6-cal_update_2*). The mean RMSD (0.018) value of the inactive compounds is at a similar level as for the internal CV on Tox21Train (*1-internal_CV*) as shown in Fig. 8b. The RMSD values of the active compounds vary more between the different endpoints. This may be explained by the small number of active compounds available in the calibration and test sets for some endpoints. To summarise, the CP models trained on Tox21Train were internally well calibrated (*1-internal_CV*) but showed poorer calibration for the prediction of Tox21Score (*2-pred_score*). Applying SCP (*3-pred_score_SCP*) or updating the training set with Tox21Test (*4-train_update*) did not improve the calibration to the same extent as when exchanging the calibration set only (*5-cal_update, 6-cal_update_2*).

### Impact of data size on the calibration

Importantly, the proofs on CP validity are made assuming an asymptotic number of test examples (i.e. requiring an infinite number of test examples) [24]. Hence, the poor calibration is not necessarily only due to exchangeability issues (or the use of ACP, for which there are no validity guarantees). The calibration could also be affected by the statistical variation due to finite test sets in all computational experiments. In the broadest sense, also the overconservative validity could be due to the finite number of test examples.

Looking at the outliers in the RMSD (Fig. 8), they mainly arise from endpoints NR_AR_LBD, NR_AR and SR_ATAD5, which are, besides NR_PPAR$\gamma$, the endpoints with the smallest overall number of actives (in all three Tox21 datasets combined). For the NR_AR and NR_AR_LBD datasets, the predictive performance (both in validity and efficiency) is expected to be less good for the active compounds, as the number of available active compounds is very small (i.e. 3 and 4 in Tox21Test and 11 and 8 in Tox21Score, respectively). If we have only eight compounds in the calibration set, this means that only nine different p-values can be obtained for a new active compound. This low resolution obviously makes it impossible to obtain perfect calibration. Since it is difficult to define a minimum required number of actives, and since the resolution for the p-values of the inactive compounds is much higher, results for all endpoints were included in the evaluation. The calibration might generally improve if the experiments were repeated on larger and/or more balanced datasets.

Although, the composition of the three Tox21 datasets may not conform with all model assumptions, this may more closely resemble many real-life scenarios where data is generated at different time points and older data is often used to predict new outcomes. All the more, it is therefore important to have strategies to improve the calibration and thus the application of CP models on new data.

## Conclusions

In this work, the potential of CP to diagnose data drifts in toxicity datasets was investigated on the Tox21 data. Deviations between observed and expected error rates was monitored using calibration plots and quantified using the RMSD from the expected calibration level. Poor calibration was observed for models trained on Tox21Train and predictions made on Tox21Score, indicating the presence of drifts between the two datasets. The distribution of the data may not be the only reason for error rate deviations in the calibration plot. In additional experiments using the newly introduced SCP framework, it was ruled out for 10 endpoints that the employed CP aggregation method (ACP) has a major impact. A second influencing factor on the calibration can be the small data set size. It was discussed that the calibration may be improved to some extent by having larger datasets, especially containing more active compounds, for model training, calibration and testing. Overall, it was concluded that the three Tox21 datasets likely do not originate from the same distribution and may be challenging for ML methods. Nonetheless, these datasets do reflect outcomes that may occur in experimental screening scenarios.

Two different model update strategies using the intermediate Tox21Test data were investigated with the aim to improve the poor calibration. The calibration of predictions on Tox21Score could be slightly enhanced by updating the training set with more recent data (Tox21Test) and retraining the models—the more natural behaviour if new data has been obtained. However, exchanging only the calibration set with newer data (Tox21Test) led to a slightly smaller error rate, albeit often with a reduction in efficiency. As an additional advantage of the *5-cal_update* strategy, retraining of the model is not required.

## Supplementary Information

**Additional file 1: Table S1.** Number of compounds available per Tox21 dataset and endpoint before standardisation. **Table S2**. Mean ± standard deviation values over all twelve endpoints for observed error rate and efficiency at SL 0.2 for all experiments.** Figure S1.*** 1-internal_CV*: ACP models were trained and calibrated on Tox21Train and internally validated.** Figure S2.*** 2-pred_score*: ACP models were trained and calibrated on Tox21Train and predictions were made for Tox21Score.** Figure S3.*** pred_test*: ACP models were trained and calibrated on Tox21Train and predictions were made for Tox21Test.** Figure S4.*** 3-pred_score_SCP*: SCP models were trained on Tox21Train and predictions made for Tox21Score.** Figure S5.*** 4-train_update*: The training set from Tox21Train was updated with Tox21Test.** Figure S6.*** 5-cal_update*: ACP models were trained on Tox21Train and calibrated on Tox21Test.** Figure S7.*** 6-cal_update_2*: ACP models were trained on Tox21Train and calibrated on 50% of Tox21Score.** Table S3.** Mean RMSD values over all 12 endpoints, calculated for all compounds, as well as for active and inactive compounds, separately.

## Data Availability

A GitHub repository with supplementary information is available under https://github.com/volkamerlab/cptox21_manuscript_SI. In the repository, the signature fingerprints for all pre-processed datasets are provided, as well as the output evaluation files from all experiments, which contain the underlying data for the CEPs and boxplots. The repository, also contains example code to demonstrate how the different ACP experiments were performed. For the SCP code, the reader is referred to the original SCP repo by Gauraha [44, 47].

## Abbreviations

ML: Machine learning
SVM: Support vector machine
EP: Endpoint
NR_AhR: Aryl hydrocarbon receptor
NR_AR: Androgen receptor, full length
NR_ER: Estrogen receptor, full length
NR_AR_LBD: Androgen receptor, ligand-binding domain
NR_ER_LBD: Estrogen receptor, ligand-binding domain
NR_PPAR_$\gamma$: Peroxisome proliferator-activated receptor gamma
SR_ARE: Nuclear factor (erythroid-derived 2)-like 2/antioxidant responsive element
SR_HSE: Heat shock factor response element
SR_MMP: Mitochondrial membrane potential
SR_ATAD5: ATAD5
NR_Aromatase: Aromatase
SR_p53: p53
CP: Conformal prediction
ICP: Inductive conformal predictor
ACP: Aggregated conformal prediction
SCP: Synergy conformal prediction
nc: Nonconformity score
RMSD: Root-mean-square deviation
Tox21Train: Training data for the Tox21 Data Challenge
Tox21Test: Test data for the Tox21 Data Challenge
Tox21Score: Final scoring data for the Tox21 Data Challenge
CEP: Calibration and efficiency plot
CV: Cross-validation
AD: Applicability domain
