# Supplementary material for: Assessing the calibration in toxicological in vitro models with conformal prediction
Source: J Cheminform. 2021 Apr 29;13:35. doi: 10.1186/s13321-021-00511-5 (PMC8082859; doi:10.1186/s13321-021-00511-5)
Supplement: Supplementary file 1 — Additional file 1: Table S1. Number of compounds available per Tox21 dataset and endpoint before standardisation. Table S2. Mean ± standard deviation values over all twelve endpoints for observed error rate and efficiency at SL 0.2 for all experiments. Figure S1. 1-internal_CV: ACP models were trained and calibrated on Tox21Train and internally validated. Figure S2. 2-pred_score: ACP models were trained and calibrated on Tox21Train and predictions were made for Tox21Score. Figure S3. pred_test: ACP models were trained and calibrated on Tox21Train and predictions were made for Tox21Test. Figure S4. 3-pred_score_SCP: SCP models were trained on Tox21Train and predictions made for Tox21Score. Figure S5. 4-train_update: The training set from Tox21Train was updated with Tox21Test. Figure S6. 5-cal_update: ACP models were trained on Tox21Train and calibrated on Tox21Test. Figure S7. 6-cal_update_2: ACP models were trained on Tox21Train and calibrated on 50% of Tox21Score. Table S3. Mean RMSD values over all 12 endpoints, calculated for all compounds, as well as for active and inactive compounds, separately. [file 13321_2021_511_MOESM1_ESM.pdf]

Additional file

Assessing the Calibration in Toxicological in Vitro Models with Conformal Prediction

Andrea Morger, Fredrik Svensson, Staffan Arvidsson McShane, Niharika Gauraha, Ulf Norinder, Ola Spjuth and Andrea Volkamer

Table S1: Number of compounds available per Tox21 dataset and endpoint before standardisation.

	Tox21Train		Tox21Test		Tox21Score	
endpoint	actives	inactives	actives	inactives	actives	inactives
NR_AhR	950	7214	30	241	73	537
NR_AR	380	8977	3	288	12	574
NR_AR_LBD	303	8291	4	248	8	574
NR_Aromatase	360	6861	18	196	39	489
NR_ER	937	6756	27	237	51	465
NR_ER_LBD	446	8302	10	276	20	580
NR_PPAR_γ	222	7957	15	251	31	574
SR_ARE	1097	6067	47	186	93	462
SR_ATAD5	338	8748	25	246	38	584
SR_HSE	428	7718	10	256	22	588
SR_MMP	1142	6174	38	199	60	483
SR_p53	537	8092	28	240	41	575

Table S2: Mean ± standard deviation values over all twelve endpoints for observed error rate and efficiency at SL 0.2 for all experiments.

nr.	name	error rate at SL 0.2	efficiency at SL 0.2
1	*internal_CV*	0.17 ± 0.01	0.77 ± 0.13
2	*pred_score*	0.31 ± 0.12	0.72 ± 0.14
–	*pred_test**	0.26 ± 0.11	0.70 ± 0.15
3	*pred_score_SCP*	0.27 ± 0.12	0.73 ± 0.13
4	*train_update*	0.23 ±0.06	0.71 ±0.15
5	*cal_update*	0.21 ± 0.05	0.51 ± 0.18
6	*cal_update_2*	0.18 ± 0.01	0.50 ± 0.17

**pred_test*: the CV-models from *internal_cv* were used to make predictions on Tox21Test.

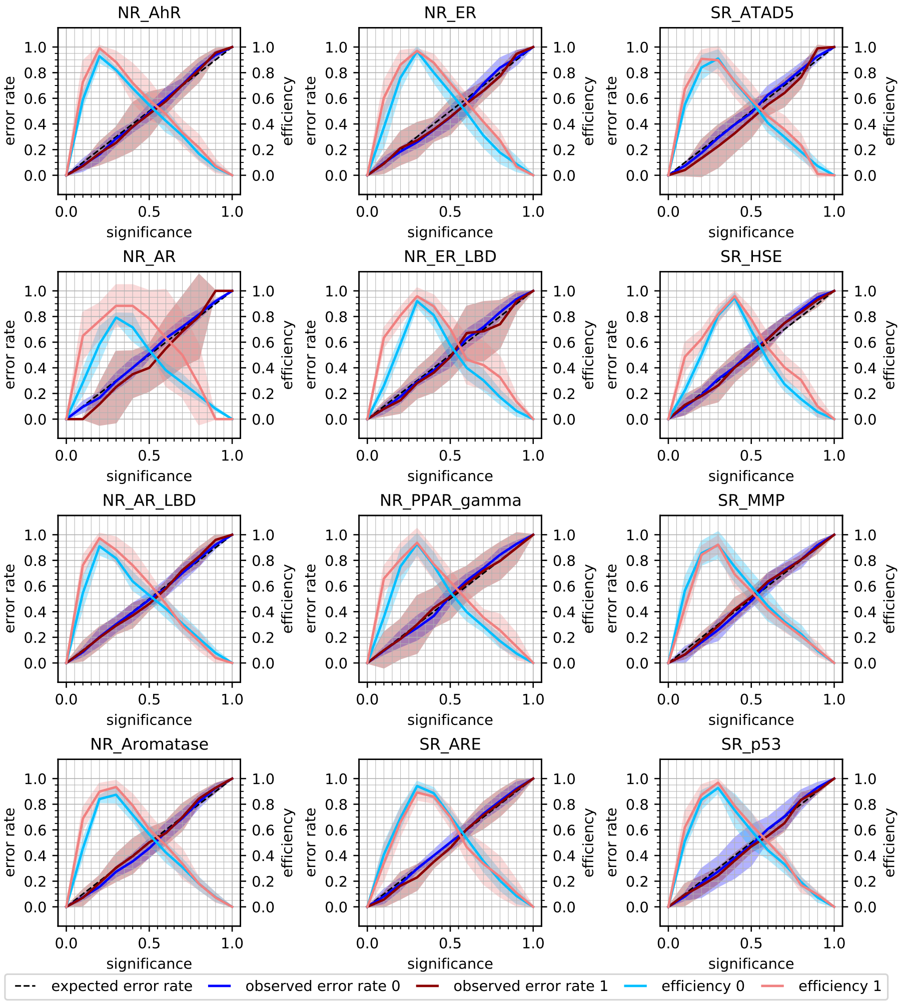

Figure S1: *1-internal_CV:* ACP models were trained and calibrated on Tox21Train and internally validated. CEPs for all twelve Tox21 endpoints are shown. Class 0: inactive compounds, class 1: active compounds. For a detailed explanation of all the components in the CEP, see Figure 2.

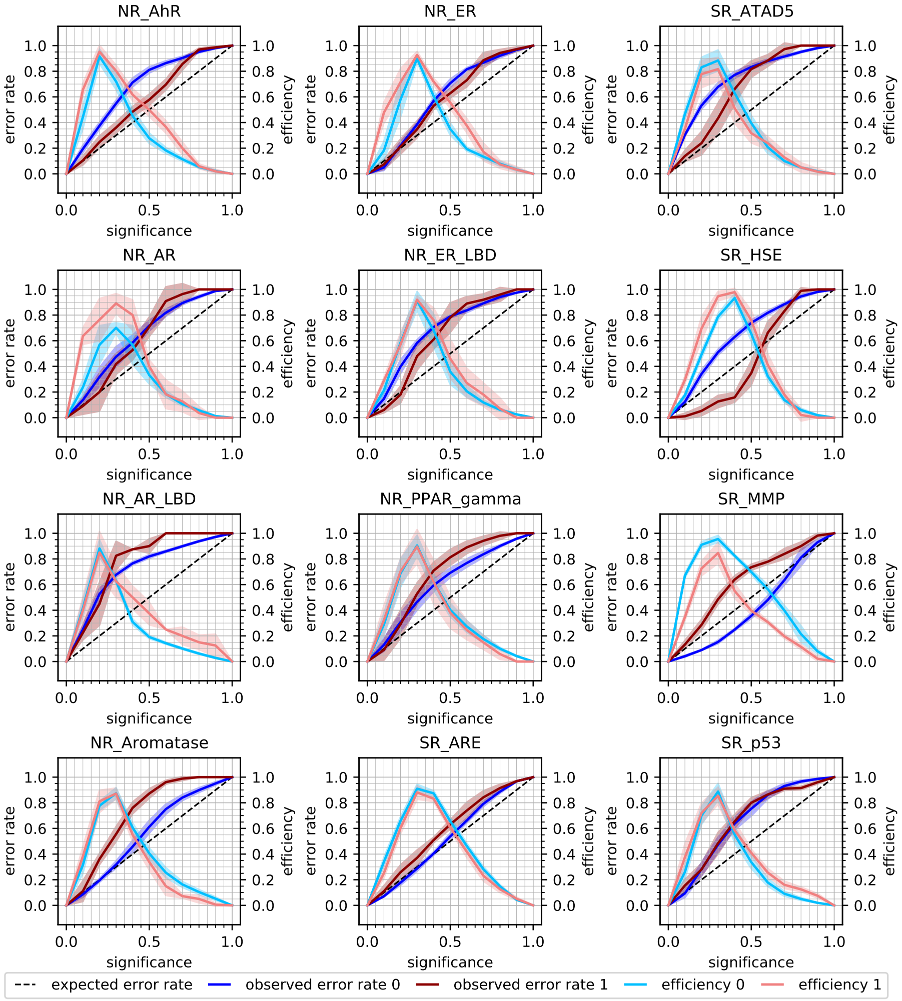

Figure S2: *2-pred_score:* ACP models were trained and calibrated on Tox21Train and predictions were made for Tox21Score. CEPs for all twelve Tox21 endpoints are shown. Class 0: inactive compounds, class 1: active compounds. For a detailed explanation of all the components in the CEP, see Figure 2.

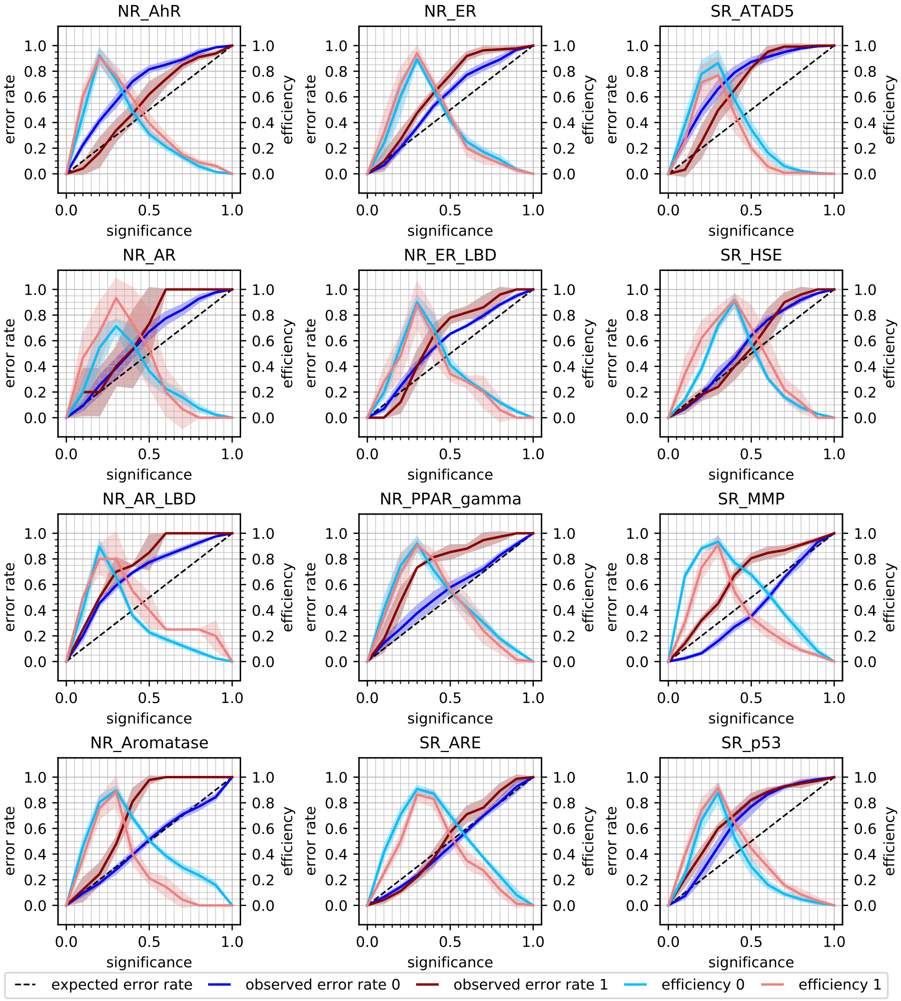

Figure S3: *pred_test:* ACP models were trained and calibrated on Tox21Train and predictions were made for Tox21Test. CEPs for all twelve Tox21 endpoints are shown. Class 0: inactive compounds, class 1: active compounds. For a detailed explanation of all the components in the CEP, see Figure 2.

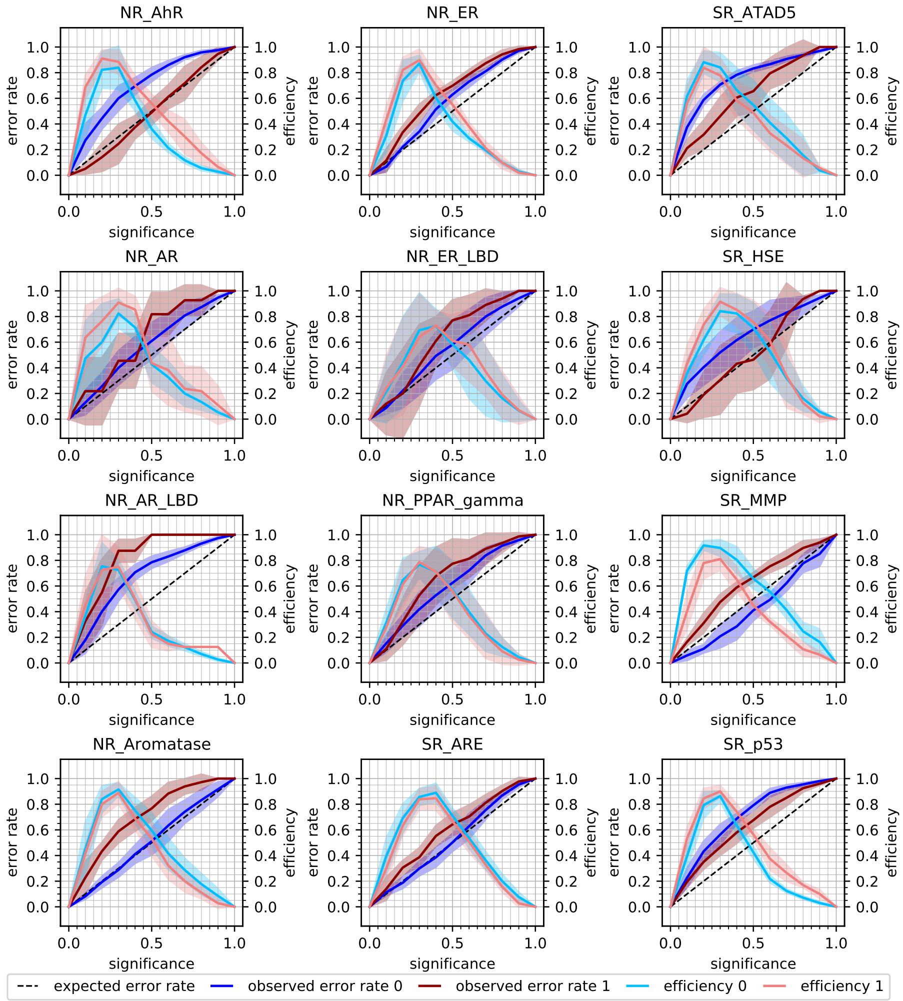

Figure S4: *3-pred_score_SCP:* SCP models were trained on Tox21Train and predictions made for Tox21Score. CEPs for all twelve Tox21 endpoints are shown. Class 0: inactive compounds, class 1: active compounds. For a detailed explanation of all the components in the CEP, see Figure 2.

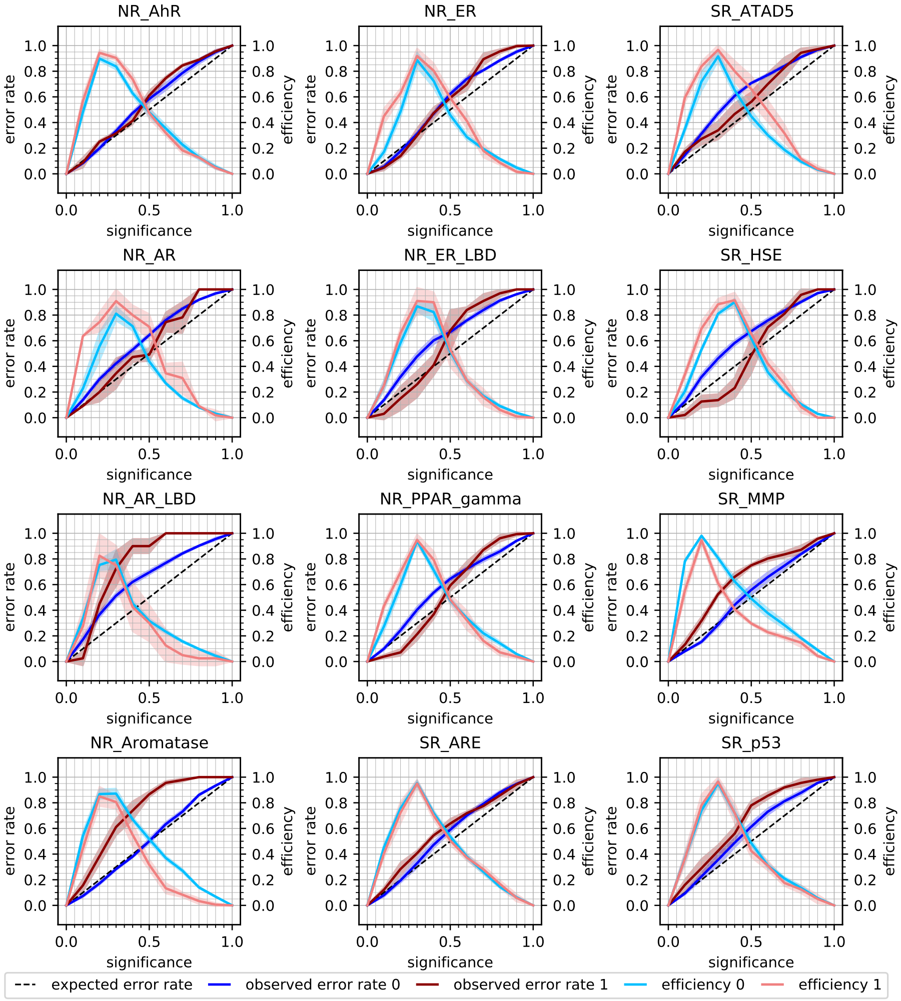

Figure S5: *4-train_update:* The training set from Tox21Train was updated with Tox21Test. An ACP model was retrained and predictions were made for Tox21Score. CEPs for all twelve Tox21 endpoints are shown. Class 0: inactive compounds, class 1: active compounds. For a detailed explanation of all the components in the CEP, see Figure 2.

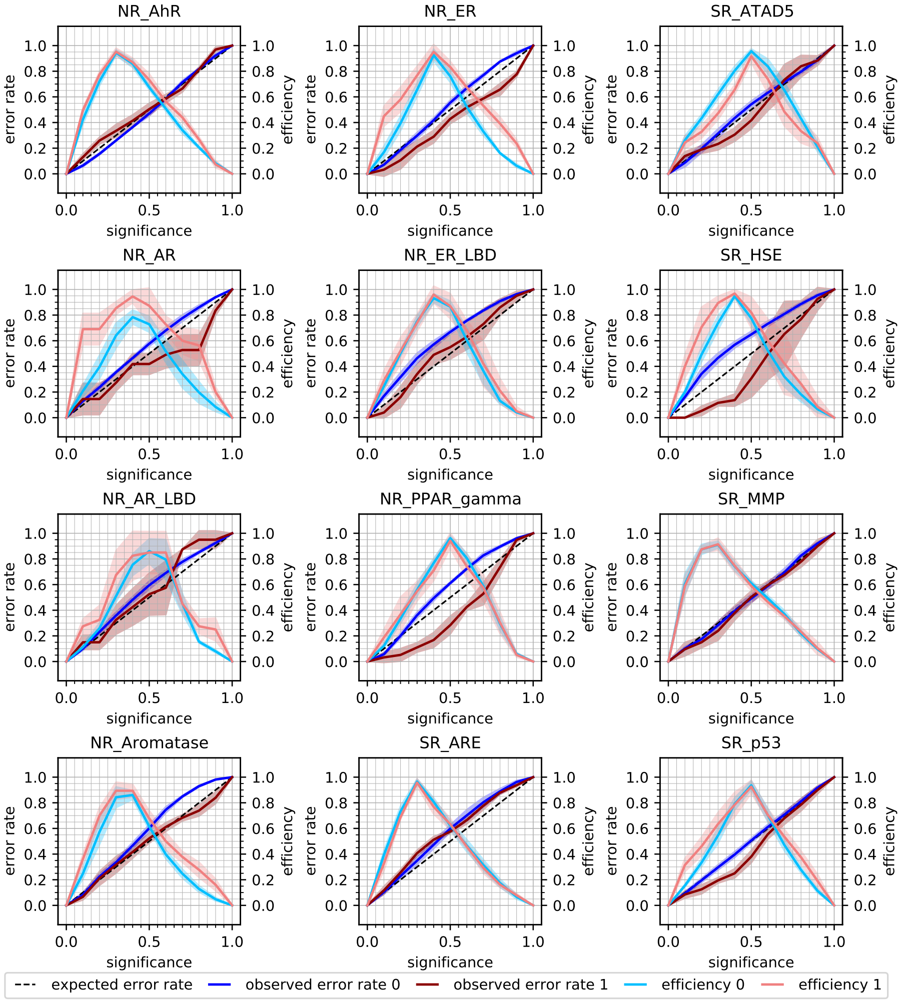

Figure S6: *5-cal_update:* ACP models were trained on Tox21Train and calibrated on Tox21Test. Predictions were made for Tox21Score. CEPs for all twelve Tox21 endpoints are shown. Class 0: inactive compounds, class 1: active compounds. For a detailed explanation of all the components in the CEP, see Figure 2.

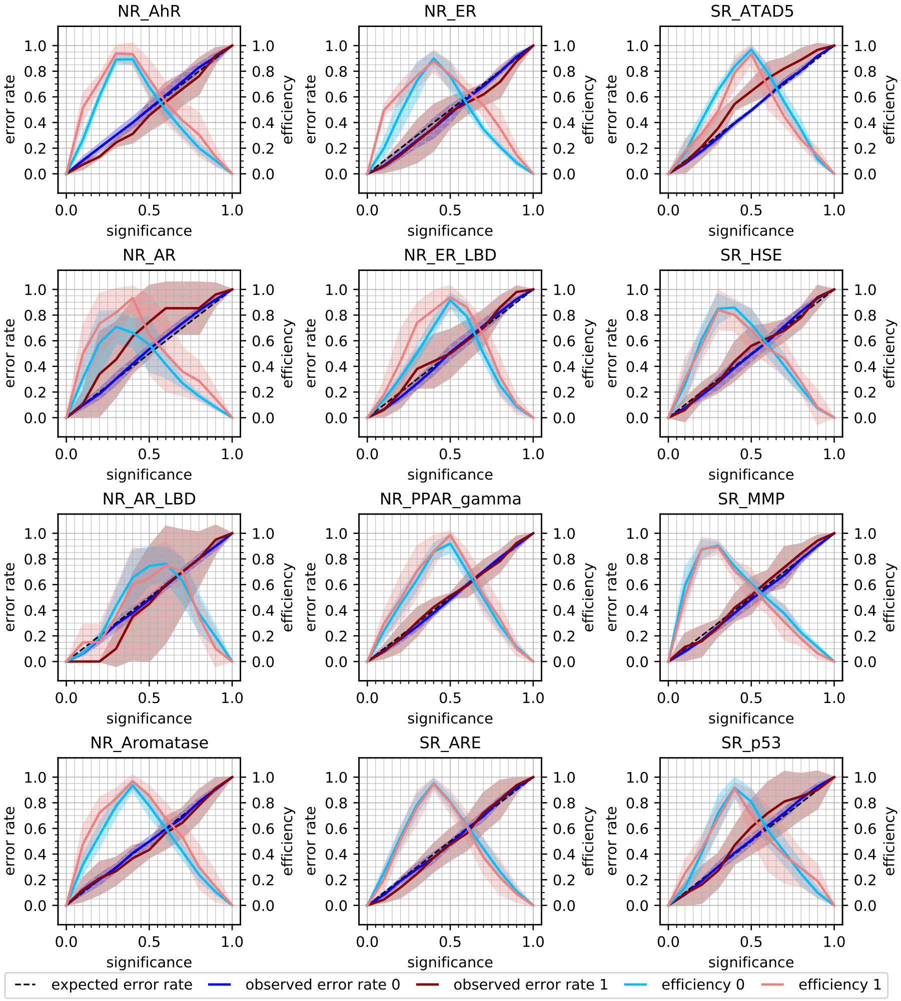

Figure S7: *6-cal_update_2:* ACP models were trained on Tox21Train and calibrated on 50% of Tox21Score. Predictions were made for the other 50% of Tox21Score. CEPs for all twelve Tox21 endpoints are shown. Class 0: inactive compounds, class 1: active compounds. For a detailed explanation of all the components in the CEP, see Figure 2.

Table S3: Mean RMSD values over all 12 endpoints, calculated for all compounds, as well as for active and inactive compounds, separately.

nr.	name	all	actives	inactives
1	*internal_CV*	0.022	0.032	0.022
2	*pred_score*	0.150	0.167	0.154
–	*pred_test**	0.116	0.180	0.119
3	*pred_score_SCP*	0.121	0.147	0.124
4	*train_update*	0.090	0.135	0.089
5	*cal_update*	0.054	0.073	0.058
6	*cal_update_2*	0.018	0.057	0.018

**pred_test*: the CV-models from *internal_cv* were used to make predictions on Tox21Test.
